# Systematic Review of Poly-4-Hydroxybutyrate: A Swiss Army Knife in Modern Breast Surgery

**DOI:** 10.1093/asjof/ojaf018.003

**Published:** 2025-05-13

**Authors:** Nicholas Vernice, Carter Boyd, Chris Amro, Thomas Sorenson, Kshipra Hemal, Jenn Park, Oriana Cohen, Nolan Karp, Mihye Choi

**Affiliations:** NYU Langone Health, New York, NY; NYU Langone Health, New York, NY; NYU Langone Health, New York, NY; NYU Langone Health, New York, NY; NYU Langone Health, New York, NY; NYU Langone Health, New York, NY; NYU Langone Health, New York, NY; NYU Langone Health, New York, NY; NYU Langone Health, New York, NY

## Abstract

**Goals/Purpose:**

Breast procedures, for both reconstructive and aesthetic indications, persistently are subject to the effects of gravity. While various surgical techniques have emerged to prevent ptosis following these procedures, it remains a persistent challenge. To provide additional support for the weight of the native breast or an implant, plastic surgeons have utilized soft tissue support as an adjunct in reducing ptosis. Many soft tissue support implants have emerged including biologic meshes, synthetic meshes, and acellular dermal matrices. Poly-4-hydroxybutyrate (P4HB), a fully absorbable polymer, has found more recent applications in breast surgery. The objective of this study was to identify uses of P4HB in both reconstructive and aesthetic breast surgery reported in the literature and synthesize the available data on its outcomes and efficacy.

**Methods/Technique:**

This systematic review adhered to the Preferred Reporting Items for Systematic Reviews and Meta-Analysis (PRISMA) guidelines with a publicly available protocol registered on PROSPERO (ID 591069). PubMed (MEDLINE), EMBASE, and Cochrane databases were searched for publications describing use of P4HB in breast surgery. Searches included all heading and keywords for the phrases: “P4HB,” “Poly-4-hydroxybutyrate,” “GalaFlex,” inclusive of the phrases “breast,” “surgery,” “reconstruction,” “implant,” “augmentation,” “operation,” or “procedure.” No language, article type, or publication date restrictions were included in the initial searches. All studies were reviewed by two independent reviewers (NV and CA). If there were any disagreements, CB reviewed the study and made the final decision regarding study inclusion. Inclusion criteria were (1) use of P4HB in aesthetic or reconstructive breast surgery, (2) be written in English. For articles selected for inclusion, bibliographies and citing references were screened from Scopus (Elsevier). From the included studies, cohort size, indication for P4HB, mean age, mean follow-up time, recurrence of ptosis, need for revisions, infection rates, and complication data were extracted. Due to significant heterogeneity in study designs, patient populations, and outcomes measured, meta-analytical techniques were not deemed feasible.

**Results/Complications:**

Following removal of duplicates, a total of 358 studies were included for initial review. Following title and abstract screening, 338 studies were deemed ineligible for inclusion. Full-text review was performed on a total of 20 studies. Following review, 16 articles were deemed acceptable for final inclusion. Of the articles meeting inclusion criteria, 1,420 patients were represented of which 1,021 patients were treated with P4HB. Included studies consisted of four retrospective cohort studies, nine retrospective case series, one prospective case series, and two animal studies.

P4HB was described as being used in a variety of clinical scenarios including soft tissue support in breast reconstruction (ADM-P4HB hybrid mesh, circumferential implant wrap, inferolateral sling, placement in the lateral gutter to re-establish the anterior axillary line), assistance in implant malposition, defining the medial cleavage area in breast augmentation, symmastia correction, soft tissue support for mastopexy, mastopexy augmentation, and a scaffold for nipple reconstruction.

One study reported significantly lower time to drain removal with P4HB in immediate expander-based reconstruction versus ADM, though only by three days (15 vs 18 days, p = 0.008). A second study reported significantly higher rates of capsular contracture with use of P4HB in dual-plane two-stage breast reconstruction (46/96; 47.9%) compared to dual-plane implant placement with an acellular dermal matrix (ADM) sling (42/122; 34.4%), prepectoral implant placement with no mesh support (49/161; 30.4%), or total submuscular implant placement with no mesh support (3/14; 21.4%), (p=0.02). No other studies reported significantly higher rates of capsular contracture with the use of P4HB and no other significant differences in common postoperative complications including infection, seroma, hematoma, extrusion, delayed wound healing, or palpable scaffold requiring intervention were noted between comparison groups. All prospective and retrospective case series reported high rates of patient satisfaction and mesh take as well as low rates of recurrent ptosis, implant malposition, capsular contracture, and all other common postoperative complications with the use of P4HB. Two studies were *in vivo* animal models exploring the use of P4HB as a biocompatible scaffold in nipple reconstruction in which a P4HB nipple scaffold was subcutaneously implanted on the dorsa of immunocompromised adult male rats, both of which reported that the presence of P4HB scaffold promoted the growth and retention of fibrovascular tissue in a manner best mimicking the native human nipple with higher rates of nipple projection with respect to non-scaffolded comparison groups.

**Conclusion:**

To our knowledge, this study is the most comprehensive evaluation of P4HB use in breast surgery. The data suggest that P4HB is a safe, cost-effective, and efficacious adjunct in a variety of indications. Given the two-year resorption timeline of P4HB, long-term follow up is crucial in evaluating the lasting efficacy of the polymer in supporting the weight of the native breast tissue or a breast prosthesis in preventing ptosis. Large-scale, randomized trials between P4HB and other types of soft-tissue support would be useful to determine non-inferiority or superiority of P4HB to other available options.

